# The Tree of Life Synagogue Attack: A Terrorist Radicalization Assessment Protocol‐18 Examination of Pre‐Attack Warnings and Post‐Attack Contagion and Copycat Effects

**DOI:** 10.1002/bsl.70066

**Published:** 2026-05-14

**Authors:** Molly Amman, Julia Kupper, J. Reid Meloy

**Affiliations:** ^1^ FBI (Retired), Molly Amman Threat Assessment LLC West Des Moines Iowa USA; ^2^ Independent Researcher Los Angeles California USA; ^3^ FBI Behavioral Analysis Unit San Diego Psychoanalytic Center San Diego California USA

**Keywords:** antisemitic violence, forensic linguistics, lone actor, Robert Bowers, terrorism, terrorist radicalization assessment protocol‐18, threat assessment, tree of life

## Abstract

This is a retrospective case study of an antisemitic lone actor terrorist who completed the deadliest attack against the Jewish community in American history. The analysis through the lens of the Terrorist Radicalization Assessment Protocol (TRAP‐18) finds that 72% of the warning indicators were present, including four proximal warning behaviors suggesting high or imminent risk for a targeted attack: pathway, identification, last resort, and energy burst. The case also illustrates the impact of this attack on subsequent violent events through forensic linguistic analysis of Terrorgram and other lone actors' communications. The discussion focuses upon mitigation of such lone‐actor violence, including the implementation of behavioral threat assessment and management (BTAM) teams, the use of structured professional judgment instruments (SPJ) to assess risk, the simultaneous and continuous monitoring of both online and on‐the‐ground activity of the person of concern, the location and speed of movement on the pathway to violence, and disaggregation of the motivation, risk and potential outcome in the case through scenario planning and dynamic management. The authors close with a discussion of pertinent questions for altering the macro environment, particularly online, to mitigate the risk of future attacks.

## 1 Introduction

Robert Bowers was a quiet man. The few who knew him had no idea he was planning a terrorist attack—just as he intended. To the outside world, he lived a solitary and unremarkable life. Never known for being aggressive, or outspoken beyond a small circle, he lived the life of the “gray man,” blending in, and staying apart from the memory or desire of others. When he attacked, he was on no one's radar. Charging into the Tree of Life Synagogue (TOL) in Pittsburgh, Pennsylvania, Bowers killed 11 and wounded six on October 27, 2018. Though he had hoped for many more casualties, it would nevertheless become the deadliest attack on the U.S. Jewish community in history. His motive was threefold: to stop Jews who were actively involved in facilitating immigration to the U.S.; to terrorize anyone thinking of doing the same; and to bring attention to the Great Replacement conspiracy theory. He is currently on federal death row for his crimes.

Though Bowers was careful to avoid drawing attention to himself or his behaviors before his crime, a retrospective analysis via the Terrorist Radicalization Assessment Protocol (TRAP‐18) reveals that high or imminent concern for violence would have been known prior to his attack had his pre‐attack behaviors been observed and reported by others to law enforcement. This article analyzes all relevant TRAP‐18 behaviors known to have occurred, rather than only behaviors noted at the time. As with other case studies using the TRAP‐18 (e.g., C. S. Allely et al. 2024; C. Allely et al. 2026; Collins and Clark 2021; Böckler et al. 2015; Ilardi et al. 2024; Kupper et al. 2024), this form of analysis furthers the goal of understanding both the behaviors that occurred and Bowers' actions to keep them secret, to facilitate more effective prevention operations in the future. The TOL attack is also noteworthy for its demonstration of the contagion and copycat effects of mass violence. An analysis of subsequent targeted attacks reveals that Bowers' crime influenced future offenders.

## 2 The Tree of Life Attack

On the morning of October 27, 2018, three separate congregations were holding services inside the TOL when Bowers pulled into the parking lot and maneuvered into a disabled parking space. He was equipped with an AR‐15 rifle, a shotgun (which he left in his vehicle), and three Glock handguns; ammunition for his firearms; hearing and eye protection; and an alarm clock of sorts intended for use as a distraction device for law enforcement if needed (United States v. Bowers 2023c). He entered the building at approximately 9:50 a.m. by shooting out a large window and opened fire on congregants (Bradbury 2018). Bowers subsequently exchanged fire with first responding officers near the entrance, retreated, and then exchanged fire with SWAT officers about 30 min later on the third floor of the building. Cornered and wounded, Bowers responded to spontaneous negotiation attempts from SWAT, giving them his name, age, and date of birth. He eventually crawled out of his hiding place and surrendered, telling officers that “all these Jews need to die” (United States v. Bowers 2023c). By the time he was in custody, 11 worshipers were dead and six people were wounded, including four police officers (Levenson and Souza 2023).

Approximately 1 minute before the attack (Morrison 2023a)—almost certainly from the parking lot—Bowers logged into his account on the Christian nationalist social media platform Gab and published his signoff: “HIAS likes to bring invaders in that kill our people. I can't sit by and watch my people get slaughtered. Screw your optics, I'm going in.” Formerly the Hebrew Immigrant Aid Society, HIAS is a Jewish humanitarian organization that assists refugees and other displaced persons with, among other things, resettlement needs. It had become Bowers' prime target in the months leading up to his attack.

### 2.1 Motivations

Robert Bowers fervently believed that there is a war on white people orchestrated by Jews, and that he was a soldier in that war (Sheehan 2023). He did not start out there, and in fact he was initially a skeptic of the idea. When he first began hearing about the “Great Replacement” conspiracy theory on talk radio espousing militant white supremacist beliefs in 2016 or 2017, he was unconvinced it was a problem of the magnitude being claimed. He began paying closer attention to the news, including mainstream news media such as CNN, MSNBC, ABC, CBS, and NBC (United States v. Bowers 2023a, 45), and doing his own research. By April 2018, he believed the *Great Replacement* was real and that Jews were behind it (United States v. Bowers 2023a, 53).

The *Great Replacement* conspiracy theory holds that white populations in the western world are being intentionally and systematically replaced with non‐white immigrants, orchestrated by (in this version of the theory) Jews. Since the coinage of this specific term, Great Replacement, in 2011 by Renaud Camus, a French novelist and conspiracist, this idea has moved from the far‐right ecosystem into mainstream discourse in Europe and the U.S. It has inspired numerous kinetic attacks including Oslo and Utoya Island, Norway, in 2011; Christchurch, New Zealand, in 2019; El Paso, Texas, U.S.A., in 2019; and Buffalo, New York, U.S.A. in 2022, just to name a few.

Upon concluding that the *Great Replacement* was a real and urgent threat in April 2018, Bowers began to plan his response. He tracked debate within the militant right community about violent versus nonviolent means of generating change, ultimately siding with proponents of violence. He considered which targets would give him the “most bang for the buck” (United States v. Bowers 2023b, 50–51). He contemplated human and institutional targets, ultimately settling on TOL after ruling out other preferred targets that were too inaccessible or invulnerable for various reasons (United States v. Bowers 2023b, 54). He chose a secondary target location not far away which was also a Jewish community facility. Bowers planned to use weapons that he already owned and had tested, having been critical of other active shooters who had failed to test and train with their equipment and, because of that, were less successful. He selected Israeli Military Industry (IMI) ammunition, appreciating the irony of killing Jews with Israeli ammunition. Among his weapons were three Glock pistols designed to shoot 0.357 magnum caliber rounds. An unusual caliber for the average Glock owner, Bowers knew 0.357 rounds to be better at penetrating body armor than more commonly encountered 0.40 or 0.45 caliber or 9‐mm bullets. This was desirable as he thought he would be likely to engage responding law enforcement officers (United States v. Bowers 2023b, 54–57).^1^ He made a least two trips to the shooting range to work on his firearm skills to ensure his tactics were at their best before the attack.

Bowers had a keen awareness of the need for operational secrecy and subscribed to the “gray man” concept—somebody who blends in and goes unnoticed and unremembered (United States v. Bowers 2023b, 58–60). He made a point of never driving past TOL in the months prior to his attack, only scouting his primary and secondary target locations in person for the first time minutes before the attack. He knew his leased vehicle had location tracking technology in it and questioned whether authorities might be looking at people who drive by sites of possible interest for violent actors (United States v. Bowers 2023b, 63–64). Deciding not to risk it, he took a calculated chance that he could find the information he needed on the internet to help him understand the TOL's location, architecture, floor plan and interior, as well as its busiest times—to ensure he had a target rich environment on the day of his attack. That calculated risk did not entirely pan out in his favor, as there were few interior photos of TOL available. Though his understanding of the building's interior was not perfect, it was good enough to allow for a successful attack. By studying TOL's internet presence, Bowers was also able to choose a day and time for the attack he thought would generate many casualties; Saturday mornings appeared to be the most active time at the synagogue.

On the morning of the attack, Bowers erased all content from his cell phone and wrote a program that destroyed the data on his computer and its six attached hard drives on a 200‐minute timer (United States v. Bowers 2023b, 64; Morrison 2023a). He hid his selected weapons and ammunition under a blanket in his car and set out. Bowers reportedly made his first scouting trip to survey TOL and his secondary target that very morning. As discussed below, upon seeing both sites he chose TOL as the primary target and attacked without further delay.

## 3 The Terrorist Radicalization Assessment Protocol (TRAP‐18)

The TRAP‐18, Version 2 (Meloy 2025) is a structured professional judgment tool developed by the third author^2^ to assist law enforcement and counterterrorism investigators prioritize cases and actively manage risk in acute situations. It is comprised of eight proximal warning behaviors (PWB) and 10 distal characteristics rationally derived from existing research on terrorism (Meloy et al. 2011; Meloy and Yakeley 2014; Meloy and Gill 2016). As predicted by the model and revealed in time sequencing analyses (Meloy et al. 2021), the PWBs occur more closely in time to a violent terrorist act than the distal characteristics, which are already in place by the time warning behaviors are observed. The PWBs have been noted to cluster among terrorist attackers rather than non‐attackers of national security concern in a multi‐dimensional scaling study (Goodwill and Meloy 2019). In particular, four PWBs in multiple studies have been found to significantly differentiate with medium to large effect sizes between targeted attackers and non‐attackers: pathway, identification, energy burst, and last resort warning behaviors (Böckler et al. 2021; Challacombe and Lucas 2018; Meloy et al. 2019). The clustering of these four proximal warning behaviors are considered indication of high or imminent risk by operational professionals within the targeted attack and counterterrorism prevention space. Published studies have found the TRAP‐18 to have excellent interrater reliability and various kinds of validity, including discriminant (does it measure only what it is supposed to measure?), content (is the coverage complete?), face (does it look right?), criterion (does it conform to accepted standards?) and postdictive (does it measure a past event's occurrence?) validity (C. S. Allely and Wicks 2022; Clesle et al. 2024). Moreover, the instrument has been found to be generalizable across extremist types including militant right, jihadist and single‐issue subjects (Meloy and Gill 2016), and is being increasingly utilized in the analysis of targeted attack risk in non‐ideologically motivated persons of concern (C. Allely et al. 2024; Allwinn et al. 2025; Meloy and Kupper 2025).

The TRAP‐18, however, is not the only structured professional judgment instrument for assessing risk of a terrorist attack. In a recent review, Brouillette‐Alarie et al. (2025) cited the TRAP‐18 and five other instruments for review: the Extremism Risk Guidance Factors (ERG22+), the Multi‐Level Guidelines (MLG‐V2), the Identifying Vulnerable People guidance (IVP), the Violent Extremism Risk Assessment (VERA), and one actuarial instrument, *Der Screener‐Islamismus*. This review only included studies published prior to December 31, 2021. Since that time, 31 additional studies have been published on the TRAP‐18 and the proximal warning behaviors, and it globally remains the most validated structured professional judgment instrument for the assessment of terrorist attack risk. Nevertheless, there are research shortcomings: the need for truly prospective risk studies, further construct validity studies, and internal consistency studies. The TRAP‐18 had the strongest postdictive effect size (pooled *r* = 0.62 [0.35–0.77], *p* = 0.0000) among the instruments tested.

Structured professional judgment instruments are clearly superior to unstructured judgment, what is legally known as *ipse dixit:* the assertion of truth based only upon personal authority. A recent meta‐analysis of structured professional judgment when directly compared to unstructured judgment in risk assessment based upon 31 studies, 169 effect sizes, and 45,673 risk judgments, found that these tools outperform unstructured judgment; findings were robust to variations in population, assessment context, and outcome measurement (Viljoen et al. 2024).

The TRAP‐18 indicators should be viewed by the assessor as patterns of risk. This is to counteract the assessor's tendency to focus on a discrete variable and guide the assessor toward a wide‐aperture view of the actor of concern. Innate human cognition for pattern recognition facilitates this technique, which finds theoretical grounding in gestalt psychology (Koffka 1921; Kohler 1929; Wertheimer 1938). The indicators are:

**Table:** 

Proximal Warning Behaviors	Distal Characteristics
Pathway	Personal grievance and/or moral outrage
Fixation	Framed by an ideology
Identification	Failure to affiliate with an extremist or other
Novel aggresion	group
Energy burst	Dependence on the virtual community
Leakage	Thwarting of occupational goals
Last resort	Changes in thinking and emotion
Directly communicated threat	Failure of sexually intimate pair bonding
	Mental disorder
	Creativity and innovation
	Criminal violence

Although this is the order in which the indicators appear in the TRAP–18 manual, that does not mean this is the order in which they are likely to emerge in a case. They can come to the attention of investigators in any order, and there are typically several PWBs in any given case. One large dataset (*N* > 500) of both lone‐actor terrorists and other non‐ideologically motivated attackers had an average of five distinct PWBs per case prior to attack, and no cases without at least one PWB (Meloy, personal communication, January 2026). However, such case studies, as the one presented here, are retrospective and include all known data in the case, including evidence only discovered in the aftermath. It is quite a different scenario than working a case in real time. Nevertheless, there is great usefulness in retrospective case analysis, as it deepens operational understanding of an individual case and contributes to the forward movement of both idiographic and nomothetic research. Post‐incident case studies drive the formation of operational insights about completed attacks, such as findings about behavioral patterns, event sequencing or other elements, and collectively provide a template for systemic data aggregation across varied agencies and professions (Kupper et al. 2023; Ilardi et al. 2024; Kupper, personal communication, March 19, 2026). Such case studies also provide a lens through which both human and AI agentic risk assessment (Acklin et al. 2025) can be conducted, and how such assessments should then be dynamically embedded (the changing risk) in management of the case by behavioral threat assessment teams.

## 4 TRAP‐18^3^ Assessment of Robert Bowers

### 4.1 Proximal Warning Behaviors

1. Pathway Warning Behavior: Pathway includes any behavior associated with research, planning, preparation for, or implementation of an attack. This indicator includes the later stages of the *pathway to violence* model devised by Fein et al. (1995) and Calhoun and Weston (2003).

This indicator is present: Bowers began planning his attack in April 2018, starting with target consideration and selection. He contemplated a range of individual and group assassinations, including the Director of the European Institute for Jewish Studies in Sweden and leaders of the U.S. Anti‐Defamation League. He eventually settled on TOL as his primary target due to Congregation Dor Hadash's support of HIAS, lack of security and proximity to him (United States v. Bowers 2023b, 54–57). He also identified a Jewish community center or Jewish federation center as a secondary attack location (United States v. Bowers 2023b, 72).^4^

In making targeting decisions during the planning stage, would‐be offenders generally assess and select options using an informal decision matrix of [a] desirability, [b] availability, and [c] vulnerability. First, the most desirable targets are typically identified based on the grievance, ideology, or cause being advanced by the planned attack. Then the subject must consider how available those targets are. Proximity is often a factor here. If an ocean must be crossed to reach the most desirable target, it will usually fall off the list at that point. Once the most available of the desirable targets have been identified, the subject will typically assess how vulnerable they are. Hardened targets with visible security are usually not attractive to the subject because of the heightened risk of failure. In this case, Bowers conducted this analysis and eventually settled on TOL as the primary target for three reasons: It was desirable due to Congregation Dor Hadash's involvement with HIAS. It was available because of its proximity to Bowers' home, only about a 25‐min drive (Turkewitz and Roose 2018). He calculated that it was vulnerable, or at least vulnerable enough, after conducting online research of the building—there was no perimeter fence or manned gate, and large glass windows offered a bankable entry point.

Bowers' online research of TOL also facilitated his choice of date and time. From a calendar posted on the website, he calculated that Saturday mornings were a very active time at the synagogue. That meant it would likely be a target rich environment. His first choice of attack date was October 20, 2018. However, Bowers determined that there would have been children at the synagogue that weekend. He later told Dr. Richard Rogers, a forensic psychologist who evaluated him before trial, that he felt the optics of killing children would not “bring more people to his cause (Souza 2023).” He therefore pushed to the next Saturday, October 27, 2018, finding that date a tidy solution because it was the last day of his vehicle lease, and he would be in custody or dead before having to pay rent for November a few days later.

He tested and trained on his chosen weapons during trips to a shooting range. He practiced marksmanship, tactical reloads, weapon handling and other tactical skills. Bowers planned to create “messy kills”—defined by Bowers as abdominal wounds—for the deterrent effect on others who might otherwise assist immigrants (United States v. Bowers 2023b, 78).

2 Fixation Warning Behavior: Fixation is an increasingly pathological preoccupation with a person or a cause, accompanied by social and/or occupational decline in functioning.

This indicator is present: Bowers spent significant time ruminating about the Jew‐facilitated invasion of illegal immigrants (United States v. Bowers 2023b, 68–90). Any topic related to Jews or immigration made him angry (United States v. Bowers 2023a, 46). He was increasingly preoccupied with the war against illegal immigrants, whom he viewed as invaders, and he was dedicated to fighting that war (United States v. Bowers 2023a, 50). Bowers reportedly shouted that “all Jews must die” while in the midst of his attack (Kim 2023). Even when it was over and the perpetrator was wounded and in custody, he told a SWAT officer he wanted all Jews to die because they were committing a genocide of his people (Ward 2018).

Bowers held an extreme overvalued belief—central to his attack—that Jews support illegal immigration in such a way as to make it an existential threat to the white population (United States v. Bowers 2023a, 681; United States v. Bowers 2023b, 10, 81). An extreme overvalued belief is a major cognitive‐affective driver of fixation (Meloy and Rahman 2020) and is “shared by others in a person's cultural, religious, or subcultural group. The belief is often relished, amplified, and defended by the possessor of the belief and should be differentiated from an obsession or a delusion. The belief grows more dominant over time, more refined and more resistant to challenge. The individual has an intense emotional commitment to the belief and may carry out violent behavior in its service” (Meloy and Rahman 2020). Extreme overvalued beliefs are simplistic, binary and absolute, and suffuse the content of all extremist ideologies.

Because he was so isolated, it seems likely that few people in the real world were positioned to note his fixation and counter his belief system. Online, however, his fixation was evident in his posting activity, particularly on Gab, a Christian nationalist platform favored by the militant right for its minimal content moderation policy and antisemitism. He made 627 posts on Gab between January 2018 when the account was created and October of 2018, the month of the attack (Kupper and Meloy 2023). His content on the platform was dominated by antisemitic ideas, much of it apocalyptic in theme. His Gab bio line read, “jews are the children of satan” (Silverstein 2018). The association of Jews as children of the devil is among the earliest of antisemitic tropes, and it is interrelated with other conspiracy theories such as blood libel and Jewish witchcraft (Reinhartz 2022).

Social and/or occupational decline was not noted, a usual element of pathological fixation. While Bowers lost his trucking job in 2017 for unclear reasons, he told one of his evaluating clinicians that he enjoyed having the extra time to relax and that he was able to live off his savings (United States v. Bowers 2023b, 36). He had no family or social circles to speak of, seeming to be satisfied with his solitary life. His only confirmed true friend had passed away in 2016 (United States v. Bowers 2022, 5).

3 Identification Warning Behavior: Identification is a psychological desire to be a pseudocommando or have a warrior mentality; closely associate with weapons or other military or law‐enforcement paraphernalia; identify with prior attackers or assassins; or to identify oneself as an agent to advance a particular cause or be belief system.

This indicator is present: Bowers saw himself as an agent to advance the cause of defending the white populace—his own people—from nothing less than extinction. In fighting back against the *Great Replacement*, he assigned himself to the role of soldier. After the attack, while in pretrial detention, he declared, “I'm being held a POW in war. There is a war on white people orchestrated by the Jew, and I'm a soldier in that war” (United States v. Bowers 2023a, 45). He considered himself a warrior (Mayo 2023) and believed people should play to their strengths, talking about his role as someone who could shoot and was not afraid to get shot (Mayo 2023). Once again it seems unlikely that others positioned to observe him would have been aware of this self‐image and the danger it represented. His isolation and concern with secrecy led him to be quite disciplined in avoiding any overt behaviors that would have given him away. Rigorous operational security is unusual among civilian mass attackers but was also noted in the Halle synagogue attacker, Stephan Balliet (Kupper, Bojsen‐Møller, et al. 2024), and the Capital Gazette mass murderer, Jarrod Ramos (Meloy and Kupper 2025).

Movement from fixation to identification is yet another red flag, as it is considered a warning that the person may be moving into operational space and mobilizing for violence (Meloy et al. 2019). In Bowers' case this transition may have occurred rapidly, though it is not entirely clear. He has described questioning the magnitude and urgency of the *Great Replacement* until he researched it himself and concluded it was real in April 2018. He has also described that he began to plan his eventual attack around the same time, suggesting that he contemporaneously embraced the *Great Replacement* conspiracy theory and viewed himself as a soldier defending his people from extinction.

4 Novel Aggression Warning Behavior: Novel aggression is an act of violence that appears to be unrelated to the intended act of concern and is committed for the first time. It is typically a test of the subject's ability to carry out a violent act.

This indicator is absent: No behaviors qualifying as novel aggression are known to have occurred. Given his long‐time interest in guns (United States v. Bowers 2023b, 62) and his ownership of 10 firearms in total (Oppel 2018), novel aggression may have been an unnecessary prelude to a shooting attack in this case.

5 Energy Burst Warning Behavior: Energy burst is an increase in the frequency or variety of any noted on‐the‐ground activities related to the target, even if the activities themselves appear benign, usually in the weeks, days, or hours before the attack. Visible social media and internet activity will likely decrease during this time.

This indicator is present: On the morning of the attack, Bowers erased the content from his mobile device. He wrote and launched a software program to ensure destruction of his computer files and those on six hard drives, deleting all data if he did not return to his keyboard within 200 minutes. He concealed his guns and ammunition under a blanket in his car, then scouted his primary and secondary targets for the first time, making note of a police station between them (Kupper and Meloy 2023). On arrival at TOL, he only saw a few people in the lobby, so he made a decision to leave ¾ of his ammunition in the car for rapid deployment to the secondary target. He logged into Gab.com from his mobile device and uploaded his final post.

**FIGURE 1:**
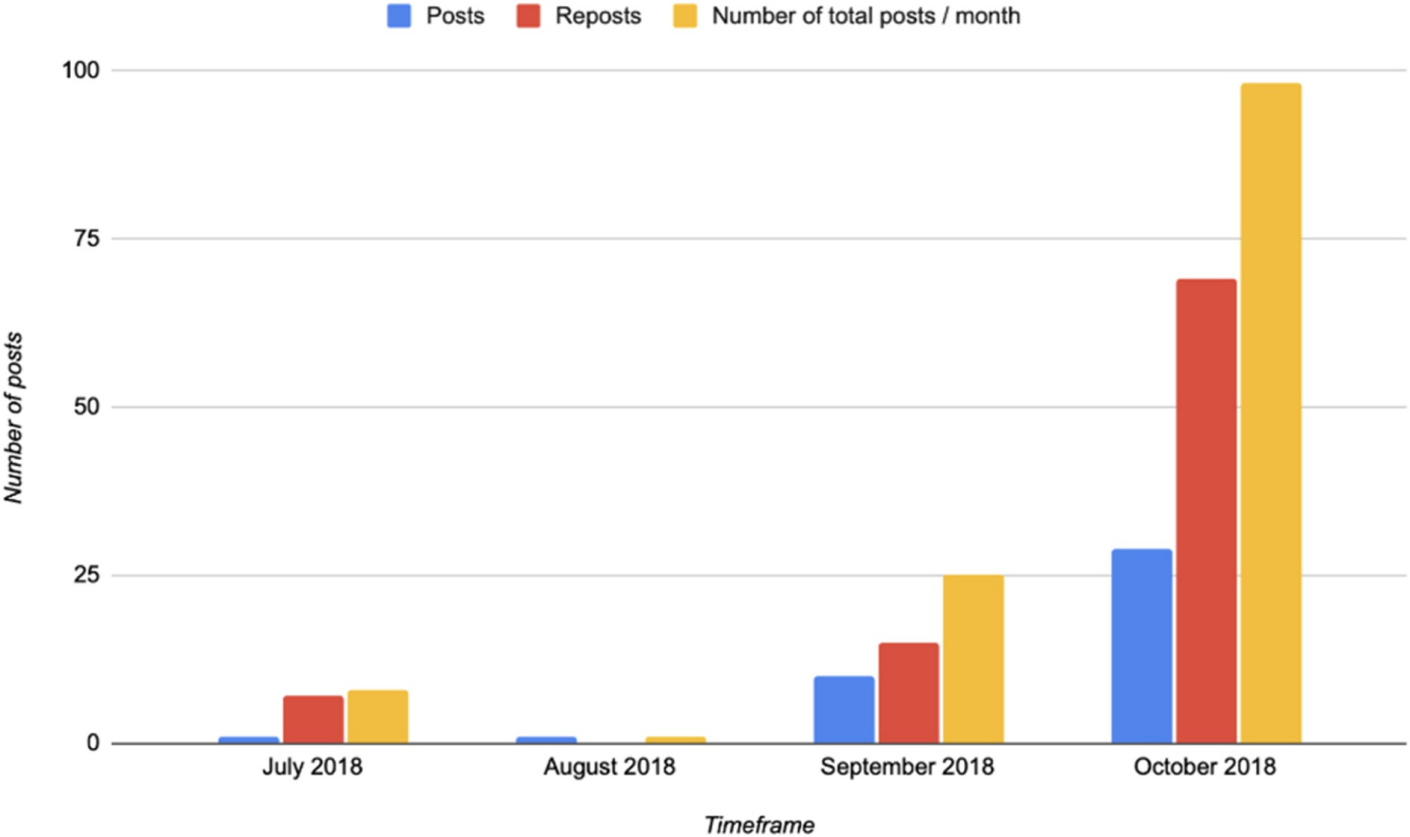
Robert Bowers' posts and re‐posts on Gab between July–October 2018. (Reprinted with permission from Kupper and Meloy 2023).

Bowers extensively posted on Gab in general. As shown in Figure 1, Kupper and Meloy (2023) chronicled an increase in his posting and reposting behaviors on the platform in the three months before the incident, with October 2018, the month of the event, marking the highest number of total posts.

**FIGURE 2:**
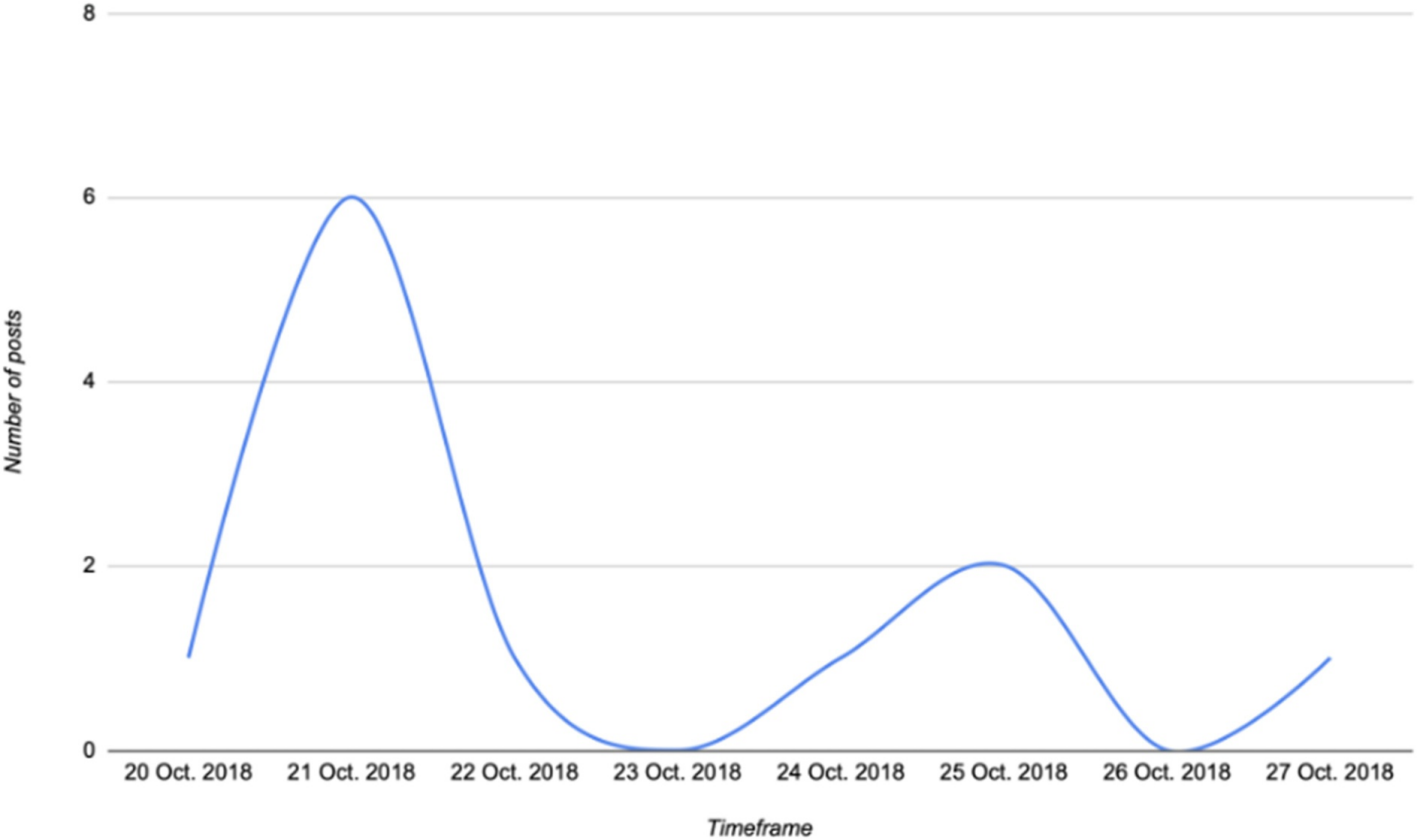
Robert Bowers' posts on Gab in the 7 days leading up to his act of violence and on the day of the attack. (Reprinted with permission from Kupper and Meloy 2023).

In the week leading up to the attack, however, Bowers' online activity decreased before he “went dark” and did not distribute any posts on the day before the attack, as shown in Figure 2. He reemerged to make his final post in the moments before entering TOL.

6 Leakage Warning Behavior: Leakage is a communication to a third party of an intent to do harm to a target through an attack; the third party may be an internet audience and/or any social media audience.

This indicator is present: Only Bowers' final post to Gab—a scant 1 minute before his attack—could be identified as leakage. Kupper and Meloy (2021) have previously argued that this posting can be classified as the perpetrator's *targeted violence manifesto*. The content of the post can be dissected as follows: communication to a third party (Gab); of an intent to do harm (“I'm going in” and “Screw your optics”); to a target (“HIAS likes to bring invaders in that kill our people”); through an attack (“I'm going in” and “Screw your optics”).

**FIGURE 3:**
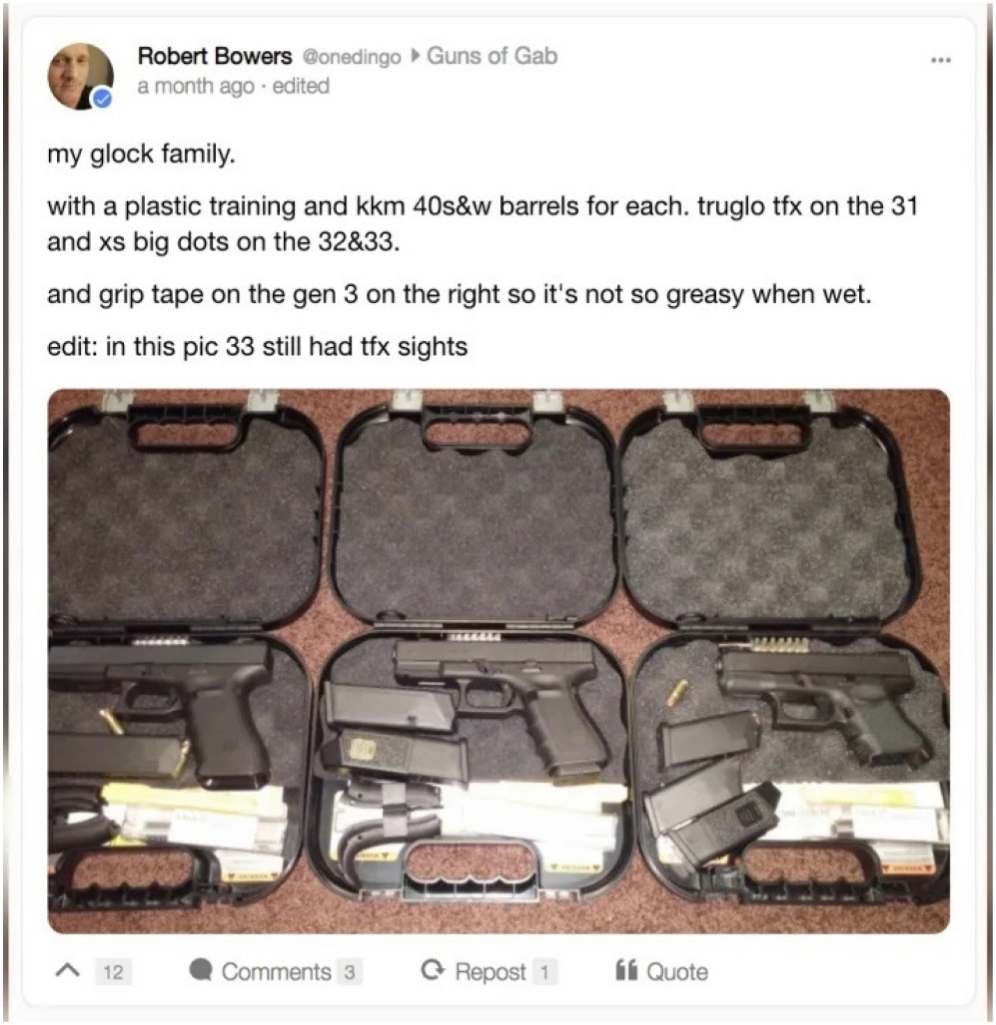
Bowers posting on “Guns of Gab,” 1 month prior to attack. (Bowers, R./@onedingo. [September 2018]. “My glock family.” www.gab.com [accessed June 12, 2023]).

Consistent with his concerns about secrecy, he was able to maintain the discipline to avoid articulating explicit violent intentions online at any point when it might have been possible to engage in meaningful threat assessment and management. Many of his posts on Gab were antisemitic, but they were not threatening. For example, in September 2018, one month prior to the incident, Bowers posted Figure 3 in “Guns of Gab,” evidencing his access to and knowledge of firearms. However, the language in this post does not suggest that these weapons would be used in a targeted violence attack:

7 Last Resort Warning Behavior: This indicator includes any evidence of a “violent action imperative” and/or “time imperative” (Mohandie and Duffy 1999); it may be a signal of desperation or distress. It is often the result of an unexpected triggering event, or one which is anticipated, that involves a loss in love and/or work. The subject believes they have no other choice and must act now.

This indicator is present: Once again, Bowers' concerns with secrecy likely contributed to the lack of evidence fitting into this category that was observable before the last moment. His final Gab post does meet criteria for last resort. The emphatic “Screw your optics, I'm going in” represent both a violent action imperative and a time imperative. “Screw your optics,” a reference to the ongoing debate within the white separatist movement between violent and nonviolent approaches, was a violent action imperative—Bowers’ declaration that he had joined the pro‐violence subgroup. “I'm going in” was a time imperative, that is, “I'm going in [now].” The situation was urgent because in his mind HIAS’ invaders were slaughtering his people. In addition, Bowers reportedly shouted that all Jews must die while in the midst of his attack (Kim 2023), what has been called a “psychological abstract” often shouted by one third of mass murderers, an affectively charged statement of motivation (Hempel et al. 1999).

8 Directly Communicated Threat Warning Behavior. This indicator is any communication of a direct threat through any means to the target or law enforcement beforehand.

This indicator is absent: No direct threats were observed, and none would be expected, given Bowers' concerns with operational secrecy.

### 4.2 Distal Characteristics

1. Personal Grievance and/or Moral Outrage. This indicator envisions the joining of both personal life experience and particular historical, religious, or political events. Personal grievance is usually composed of a major loss in love or work, feelings of anger and humiliation, and the blaming of others. Moral outrage is typically a vicarious identification with a group which has suffered, even though the lone actor usually has not experienced the same suffering.

This indicator is present: While no personal grievance was identified, Bowers harbored a strong sense of moral outrage for the perceived victimization—to the point of certain extinction—of the white population by Jews. A week before the attack he reposted a message that Western civilization was “headed toward certain extinction within the next 200 years” (Lord 2018). Although he had never actually read it, Bowers believed deeply in the content of Declaration 7 of the *White Genocide Manifesto*: “All western nations are ruled by a Zionist conspiracy to mix, overrun and exterminate the White race” (United States v. Bowers 2023a, 85). His conviction contributed to his extreme overvalued belief about Jewish/HIAS facilitation of illegal immigration and Dor Hadash's complicity. Bowers had not personally been replaced—he was and is still here. But he vicariously identified with the perceived victim group—his own people—in anticipation of the day when the replacement is irrevocable.

**FIGURE 4:**
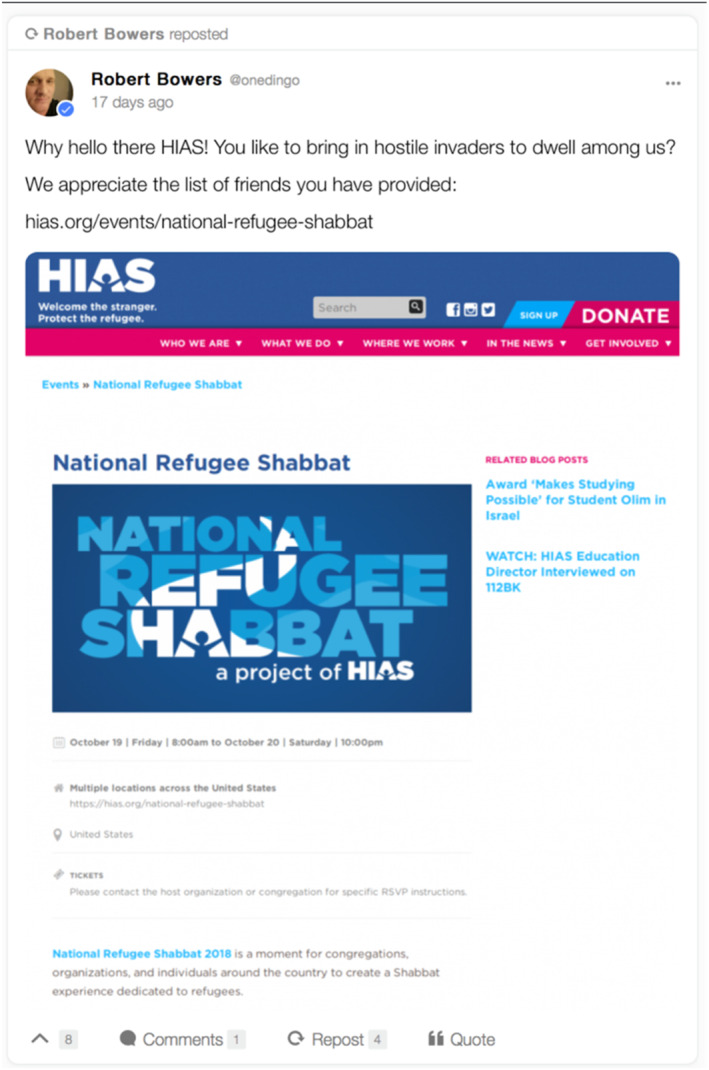
Bowers' posting on Gab 2 weeks prior to attack. (Bowers, R./@onedingo. [October 2018]. “Why hello there HIAS.” www.gab.com [accessed June 12, 2023]).

For example, approximately 2 weeks prior to the attack, Bowers first mentioned his future target in a post on Gab, as shown in Figure 4: “Why hello there HIAS! You like to bring in hostile invaders to dwell among us? We appreciate the list of friends you have provided: https://hias.org/how/refugee‐shabbat/” along with an image of two events on October 19 and 20, 2018. The latter was his original planned date for the mass shooting at the TOL synagogue.

2 Framed by an Ideology. This indicator reflects the presence of beliefs that justify the subject's intent to act. It can be a religious belief system, a political philosophy, a secular commitment, a one‐issue conflict, or an idiosyncratic justification. Beliefs are typically superficial and are chosen to justify violence.

This indicator is present: Bowers subscribed to the *Great Replacement* conspiracy theory: that Jews were bringing about a catastrophic invasion of illegal immigrants. His ideology principally centered on the truth of the *Great Replacement*, that it was an existential threat to white people, that a war was ongoing to fight back against it, and that Bowers was a soldier in that war. It was a militant mishmash of negative ideas about Jews, positive ideas about white people and urgency of action. Bowers did recognize that others had culpability in the illegal immigration problem other than Jews, but he believed Jews were disproportionately involved. Accordingly, he believed, “you deal with what's the biggest problem,” meaning addressing the biggest threat first (United States v. Bowers 2023b, 51).

For example, in September 2018, Bowers posted the following message on Gab: “just want to put this psa out there for all the vile degenerate oven dodgers. The goyim know. This is becoming increasingly obvious. Eventually it will not be safe here for you and you will be unable to leave. It takes time to convert your stuff to shekels and flee. Time is critical. That is all” (Anti‐Defamation League 2018). This post not only illustrates that the Jewish community's status as the “out‐group” (by using phrases such as “vile degenerate oven dodgers”), but it is also an indicator that violence against them in his opinion was justified. In that veiled threat, Bowers wrote, “eventually it will not be safe here for you and you will be unable to leave. It takes time to convert your stuff to shekels and flee.” His “in‐group” are the goyim (referring to a non‐Jewish person), who knew (what the Jews were up to). In October 2018, Bowers increasingly posted antisemitic rhetoric that suggested killing Jews, for example, the phrase “Make Ovens 1488F Again” (Anti‐Defamation League 2018), combined with a picture of a fiery oven such as those used in Nazi concentration camps. “14” is a white supremacist reference to the 14 Words slogan coined by David Lane, “We must secure the existence of our people and a future for white children,” and “88” the well‐known numerical code for HH or “Heil Hitler” (Lane, as cited in Michael 2009, 45).

3 Failure to Affiliate with an Extremist or other Group. This indicator encompasses the experience of rejecting or being rejected by a radical, extremist, or other group with which the subject initially wanted to affiliate.

This indicator is absent: No evidence is apparent that Bowers ever had a desire to affiliate at any point in his life. It is likely that his personality construct prevented him from attempting to affiliate or desiring to do so (see below discussion of Mental Disorder).

4 Dependence on the Virtual Community. This indicator involves use of *any* cyberspace for virtual interaction (e.g., reinforcement of beliefs) or virtual learning (e.g., planning and preparation for an attack).

This indicator is present: Bowers was socially isolated, enjoying only superficial relationships with family and acquaintances, leaving him to drive his energies toward the digital sphere. He spent significant quantities of time online, developing his personal ideology and planning for his eventual attack. The online world was also where he expressed himself, such as posting and reposting propaganda and other antisemitic material on Gab.

For example, one of Bowers' pre‐trial evaluators noted, “He states that he had always been right‐wing and conservative leaning, but became much more focused on Jews beginning in 2017–2018. He states that as he was not driving trucks at the time, he could spend more time investigating claims he had been hearing for years about how “Jews are running the media” or “Jews are controlling the country.” He states these claims were becoming “louder” and more mainstream, prompting him to investigate further. He would find links under the comments section in Youtube [*sic*], and increasingly became more and more convinced about the accuracy of such claims. He joined GAB, a far‐right social media website, which further provided support and evidence for these beliefs. He states he became aware of Jewish controlled efforts to attack predominately white countries by encouraging immigration, a plan he referred to as the “Great Replacement”” (United States v. Bowers 2023d, 10).

5 Thwarting of Occupational Goals. This indicator reflects a major setback or failure in a planned academic and/or occupational life course.

This indicator is absent: Bowers had become unemployed somewhat recently as a long‐haul trucker, though the reason for it is not known. He reported being quite content with unemployment, as it gave him time to relax and investigate claims he was hearing about Jews. He was able to live off his savings. There is no evidence that he was angry or humiliated about losing that job or being unemployed.

6 Changes in Thinking and Emotion. This indicator focuses on three areas for the assessor: changes in interpersonal style of communicating with others; changes in internal fantasy (often apparent through social media postings); and/or changes in emotional states toward those they consider unbelievers or enemies.

This indicator is present: Bowers had an interest in right‐wing and some far‐right political views in the years leading up to 2018 (Lord 2018). However, he experienced intensified anger while researching claims about Jews, concluding that everything he heard about them and the *Great Replacement* on far‐right channels was true. His prior disdain and dislike for non‐whites devolved to anger, contempt, and disgust (Matsumoto et al. 2017) for them (e.g., “jews are the children of satan” on his Gab bio line, as referenced above). Bowers tracked the debate within the white supremacist community about optics and concluded that violent action was necessary to ward off the threat of extinction. He became a warrior for his people, determined to go to war potentially at the cost of his own life. Those who remember Bowers from an earlier time in his life were shocked at the change in his ideas, once they heard he was the TOL attacker (Lord 2018).

7 Failure of Sexually Intimate Pair Bonding. This indicator is present when, over the course of the person's life as an adult (18+ years), there is *no* evidence of a sexually intimate relationship with someone for whom there is *also* a positive attachment or bond that has endured for a period of time.

This indicator is present: Bowers was never married and had no children. He described having had a few girlfriends, though an evaluating psychiatrist concluded they lacked any real depth of relationship. Fundamentally, Bowers did not seem to understand what a true relationship with another person was (United States v. Bowers 2023a, 53–54). A secondary component of this indicator is the sexualization of violence. There is no evidence in the record that this occurred.

8 Mental Disorder. This indicator reflects that evidence indicates a mental disorder is or has been present in the subject.

This indicator is present: There were conflicting opinions among experts in this case about Bowers' mental health. The factual record in the case appears to most strongly support a diagnosis of Schizoid Personality Disorder (SPD) by Dr. Park Dietz, a forensic psychiatrist who interviewed the perpetrator in prison for approximately 15 hours over the course of 3 days (Morrison 2023b).

**FIGURE 5:**
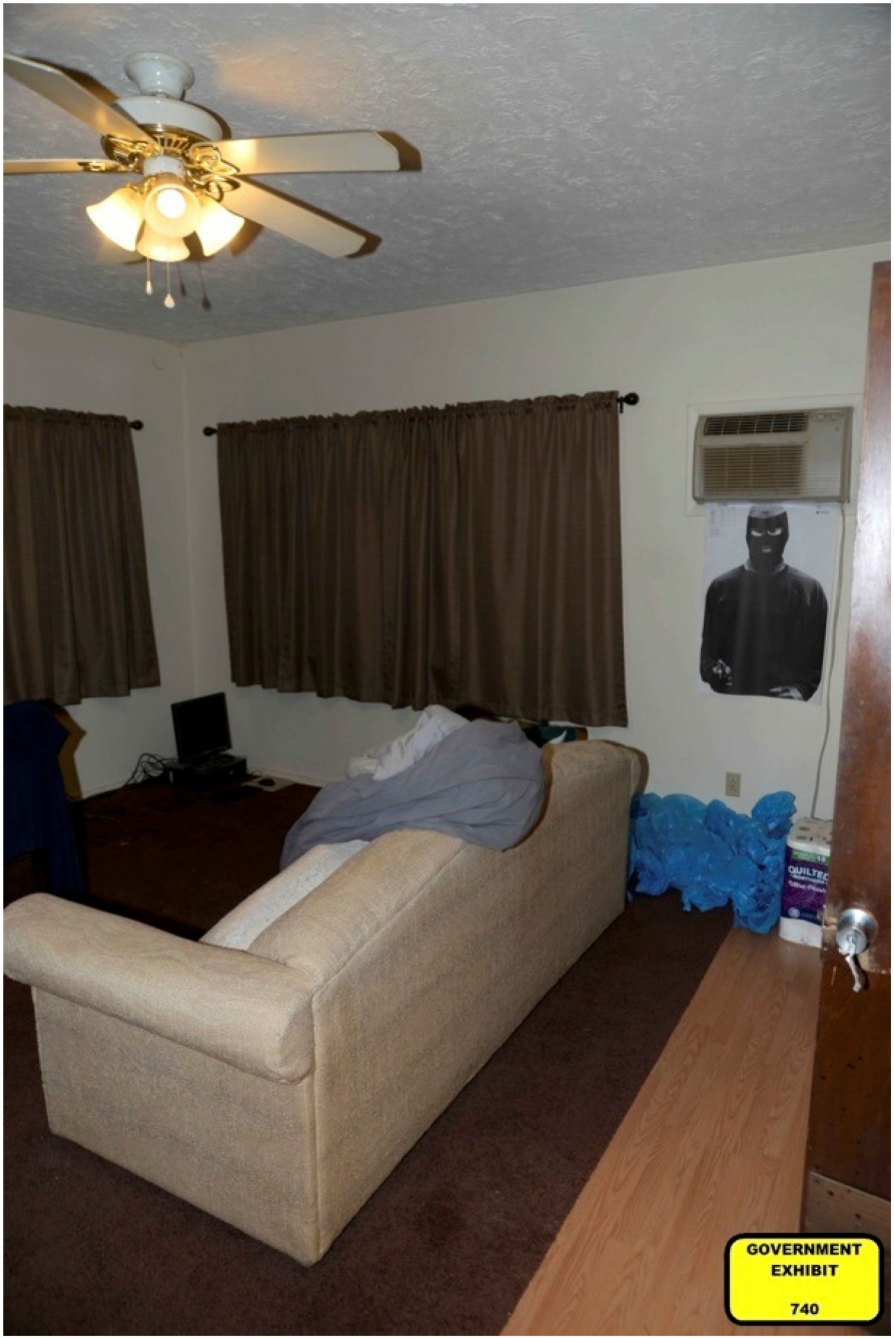
Robert Bowers apartment as photographed by law enforcement following the attack. (United States v. Bowers [2023c]. Government Exhibit 740.).

Bowers almost always chose solitary activities, he lacked close friends or confidantes beyond first‐degree relatives, he was indifferent to praise or criticism of others, and he demonstrated a flat affect most of the time. The operational significance of SPD is that it allowed Bowers to completely detach from his crime and the horrific damage it caused. For example, one of the first things he said during his jail intake on October 27, 2018, was that he would probably be there forever, and at least he would not have to worry about food. In other words, his concern was for his own well‐being and the benefit that he would not have to earn a living or fend for himself. There was no expression of emotion about what he had just done. Consistent with a diagnosis of Schizoid Personality Disorder was the post‐offense observation within his apartment that the only decor on the walls was a target, as well as a timer of some sort on his kitchen counter, as shown in Figure 5. There was a complete absence of any photographs of other human beings, including family members or friends.

9 Creativity and Innovation. This indicator reflects evidence of tactical or strategic thinking “outside the box,” meaning the planned terrorist or targeted violence act is creative (which could result in a major tactical or strategic aspect of a plan has not been done before in contemporary times) and/or innovative (the subject would be one to come up with some operational aspect likely to be imitated by others in the future).

This indicator is present: Bowers planned and carried out the most lethal attack on the Jewish community in the history of the United States. Had everything gone as planned, it could have been much deadlier. Carrying out the “biggest” attack (i.e., a maximal body count) is often celebrated within targeted violence fandoms. In 2018, Jarrod Ramos carried out the most lethal attack on journalists in the U.S. (Meloy and Kupper 2025); Seung‐Hui Cho's 2007 attack at Virginia Tech remains the deadliest of the higher education attacks (Virginia Tech Review Panel 2007); Adam Lanza, who murdered 26, mostly children, at Sandy Hook Elementary School, was an avid compiler of data on mass murders (Office of the Child Advocate 2014).

Other demonstrations of this indicator included Bowers' original plan to attack two Jewish sites in rapid succession, his focus on “messy kills” specifically for their perceived deterrent effect, and his use of IMI ammunition in appreciation of the irony of killing Jews with Israeli bullets. Bowers was also unusually clear‐headed about the role he and his attack would play. Many in the extremist white power/white separatist movements believe a race war is imminent; however, Bowers was somewhat unusual in that he correctly understood that this is not true. He recognized that he might be alone at that moment in taking up arms and he accepted that (United States v. Bowers 2023a, 96).

This item is approached differently in retrospective analysis than during a real‐time threat assessment. In real time, it could certainly be possible to obtain direct evidence of this indicator from journals or other notes made by the subject. But lacking that, the assessor thinks creatively about how this subject might carry out an attack if able to do so. Does this subject tend to think “outside the box?” is the central question for the assessor who is focused upon a person of concern (Meloy 2025).

10 Criminal Violence. This indicator is present when there is evidence of instrumental criminal violence in the subject's past, demonstrating a capacity and a willingness to engage in predation for a variety of reasons, such as a history of armed robberies or planned assaults on others for material gain.

This indicator is absent: Bowers had no criminal history of violence prior to the TOL attack.

In summary, Bowers demonstrated six of eight PWBs—all except novel aggression and directly communicated threat—and seven of 10 distal characteristics—all but failure to affiliate, thwarting of occupational goals, and criminal violence. He was positive for 72% of the TRAP‐18 indicators at the time of his attack, and exceeded the average of five PWBs. Many of these indicators were not reasonably visible to the outside world due to Bowers' intense concern with secrecy. He was very disciplined with the PWBs once he had made his decision to mobilize for violence. However, his distal characteristics were visible to others and would have merited further observation of Bowers had someone noted them (Kahneman 2011). One advantage of structured professional judgment instruments, when applied with fidelity, is that they focus on relevant correlates of risk by providing an organizing template for the observer. In the absence of such an *aide‐memoire*, professionals are prone to solely rely on their personal experience and are misled by their own cognitive biases (Kahneman 2011).

## 5 Violence Contagion and Copycat Effect of Robert Bowers

### 5.1 Targeted Violence Communications

**FIGURE 6:**
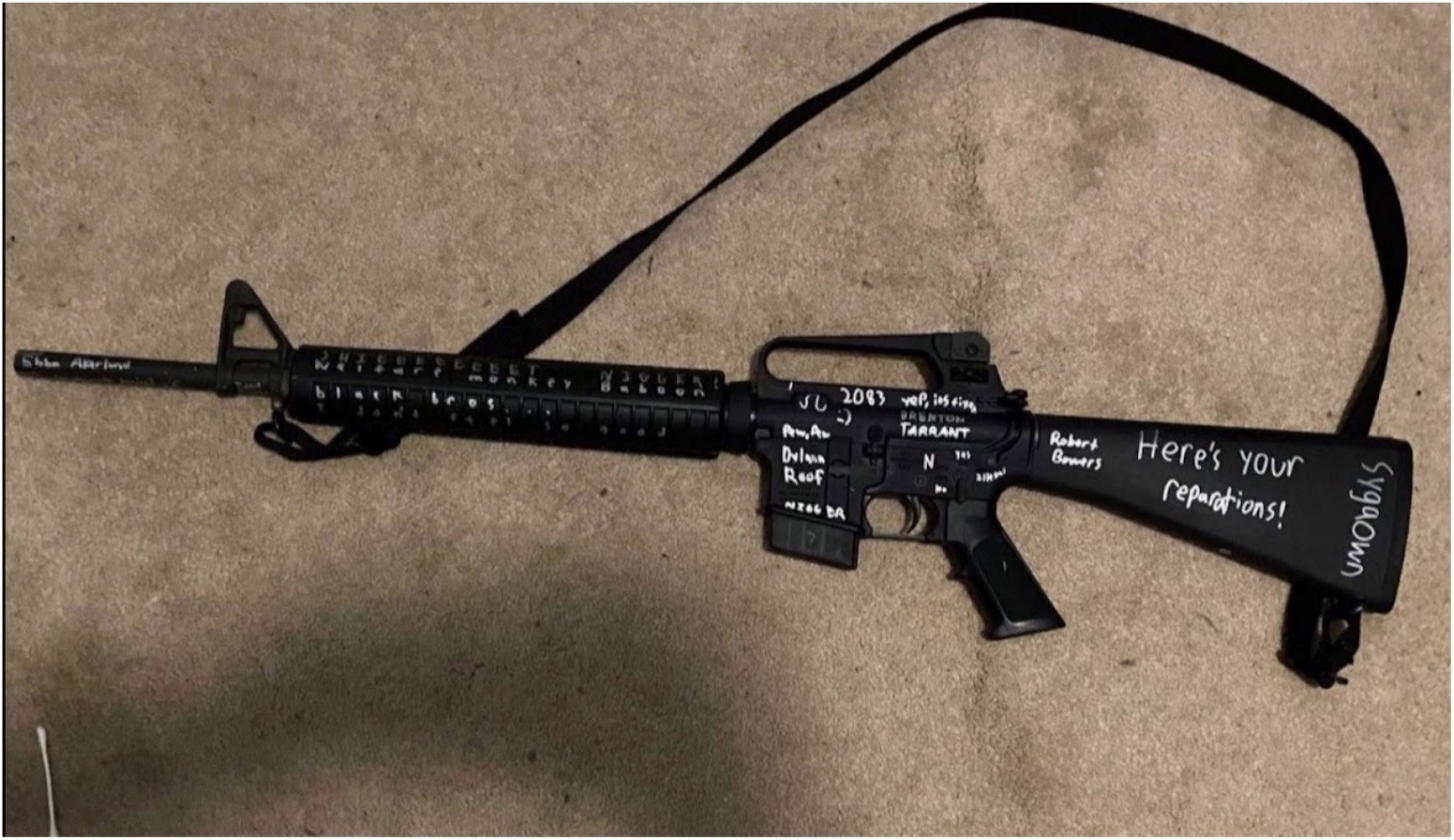
Robert Bowers' name written on Payton Gendron's firearm used during the mass shooting in Buffalo, New York, in May 2022. (As cited by Kupper and Meloy (2023)).

Robert Bowers' name and crimes have been directly referenced by numerous lone‐actor terrorists in their attack‐related communications. John Earnest, who attempted to conduct a mass shooting at a synagogue in Poway, California, in April 2019, mentioned Bowers five times in his targeted violence manifesto (Kupper et al. 2022). For instance, he wrote: “To my brothers in blood. Make sure that my sacrifice was not in vain. Spread this letter, make memes, shitpost, FIGHT BACK, REMEMBER ROBERT BOWERS” (Earnest 2019, 5). Payton Gendron, the May 2022 attacker of a supermarket in Buffalo, New York, which was frequented by black Americans, referenced Bowers in his manifesto and online log, and wrote his name on the weapon used during the shooting (as shown in Figure 6). These types of writings can have several meanings, including glorifying or preserving previous perpetrators; infusing features of animacy for an inanimate object—called object personification—to enhance emotional connections with the weapon; and/or offering a form of encouragement for future copycats (Earnest 2019).

In October 2022, Juraj Krajčík posted a tweet about his “heroes and role models,” among them Bowers, two days before committing an attack against the LGBTQIA+ community at a bar in Bratislava, Slovakia (Kupper et al. 2023). Furthermore, Bowers' name was mentioned three times in the online log of school shooter Solomon Henderson, who attempted to commit a mass casualty incident in Nashville, Tennessee, in January 2025. One example is: “My father always said you have be retarded or faggots to get caught go jail for anything not related to money. Everytime [*sic*] he said this I would think about heros [*sic*] like Brenton Tarrant, Alexander bissosne [*sic*], Ander Breivik or Robert Bowers” (Henderson 2025, 280). More indirect references to Bowers include writings on Ryan Palmeter's AR‐15, which was utilized in a shooting at a dollar store in Jacksonville, Florida, in August 2023; it stated “Fuck OPTICS!”—likely a reference to Bowers' final post on Gab (Kupper 2024). The same phrase was also written on a gun used by Robin Westman in a school shooting in Minneapolis, Minnesota, in August 2025, along with Bowers' name and other references. Lastly, Brenton Tarrant, who committed a mass shooting at two mosques in Christchurch, New Zealand, in March 2019, had named his Twitter profile picture “Screw your optics,” identical wording to Bowers' final online statement (personal communication with Chris Wilson, August 2025).

These are all examples of how lone‐actor terrorists are not just influenced and inspired by previous attackers, but how they glean tactical advice and operational strategies from them to implement a successful attack. Bowers himself claimed that he practiced with his weapons in the lead up to the crime because another synagogue shooter's weapon jammed and he wanted to avoid this scenario. Dr. Park Dietz testified during the trial that Bowers also criticized a subject who had conducted a shooting in Atlanta for not being familiar enough with his firearm. Furthermore, Bowers told one of his evaluators that he had studied the attack by Anders Breivik (United States v. Bowers 2023d), who killed 77 people and injured 319 during two consecutive attacks in Norway in July 2011.

### 5.2 Terrorgram

Most of the perpetrators discussed in the above section were part of an intricate online ecosystem and to some degree motivated by militant accelerationism, that is, “a set of tactics and strategies designed to put pressure on and exacerbate latent social divisions, often through violence, thus hastening societal collapse” (Kriner 2022). The Terrorgram community, “a loosely connected network of Telegram channels and accounts that adhere to and promote militant accelerationism” (Kriner and Ihler 2022), authored and disseminated a number of publications to incite lone actors to commit violent attacks on a global stage (Kupper and Dittrich 2024). Two of the four written propaganda pieces that were released between 2021 and 2022 referenced Bowers' name on numerous occasions, for example the first publication, *Militant Accelerationism: A Collective Handbook*. Page 24 details a terrorist “SAINTHOOD” calendar with names, kill counts, and dates of attack of several perpetrators, which includes “SAINT ROBERT GREGORY BOWERS (11) 10/27/18.” Many copycats aspire to become immortalized as martyrs in these online environments, for instance on Terrorgram, an aspiration accentuated by the often‐unsuccessful nature in their offline lives (Kupper et al. 2023, 2024; Kupper and Dittrich 2024). Additionally, the publication *Do It For The Gram: The Collected Writings of Terrorgram*, referenced Bowers four times in total. Examples include:

- P. 21: section “JEWS”
  ◦ “To commemorate Robert Bowers Day, remember that the Tree of Life synagogue was a justified military target, in which jews of all branches conspired to deprive our people of our birthright.”
- P. 73: “AVOIDING ARREST”
  ◦ “What groups were Tarrant, Bowers, Roof, McVeigh, Rudolph, Breivik or Crusius affiliated with? Exactly.”
- P. 83: “CALCULATIONS”
  ◦ “Because the System can survive a Tarrant every year, or a Bowers every couple of months. They cannot survive them every week, which is why they try to entrap those most willing to replicate these actions.”

Moreover, Bowers and his act of violence are highlighted in Terrorgram's *White Terror* documentary, released in October 2022 and depicting 106 individuals acknowledged by Terrorgram for their lone‐actor attacks (Kupper and Dittrich 2024). These traceable links of linguistic evidence highlight how Bowers' act of targeted violence has been taken up by different threat actors, with the main goal of influencing and inspiring future attackers.

**FIGURE 7:**
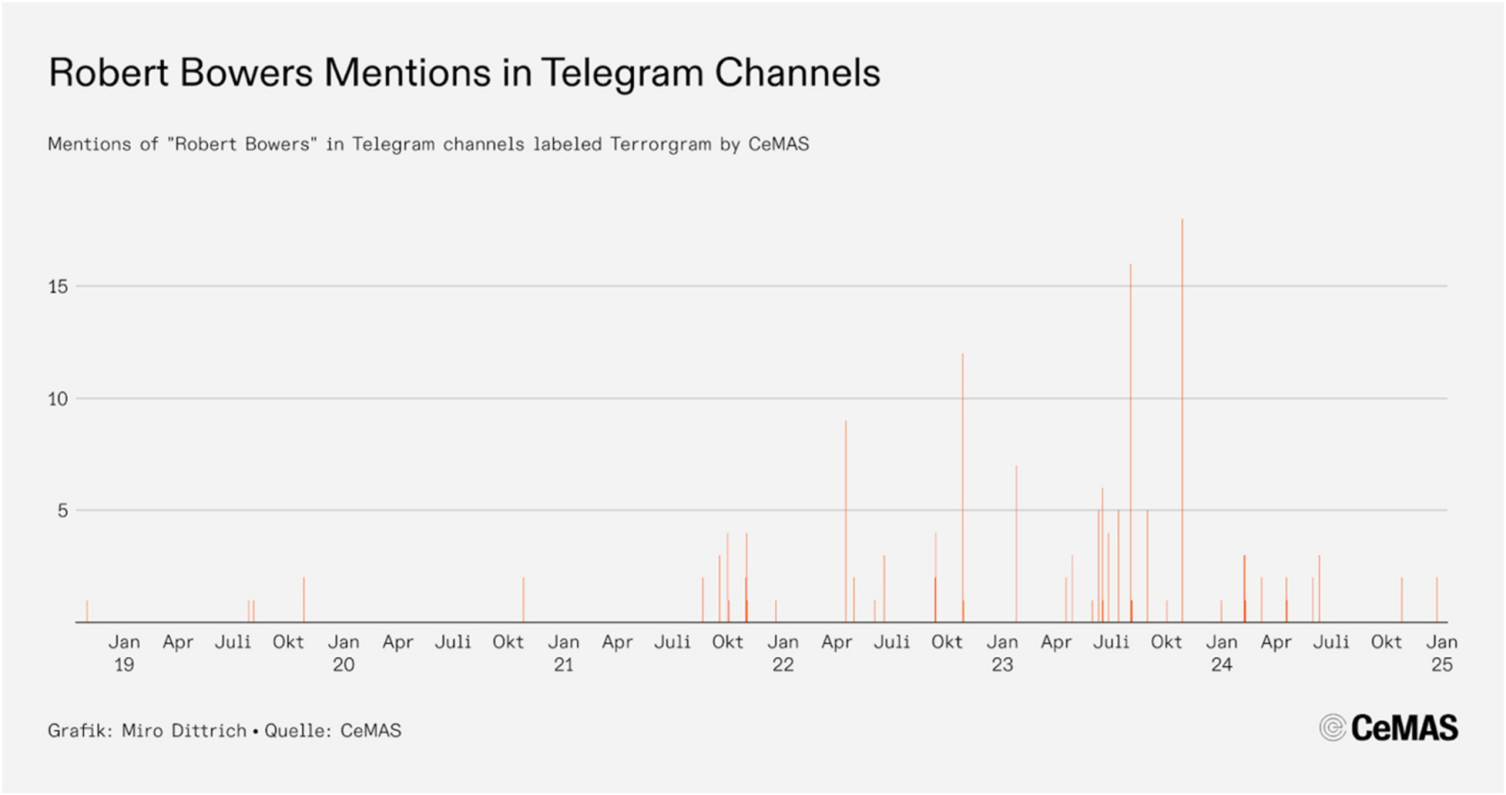
Mentions of Bowers in Telegram channels. (Personal communication with M. Dittrich 2025; graphic used with permission).

**FIGURE 8:**
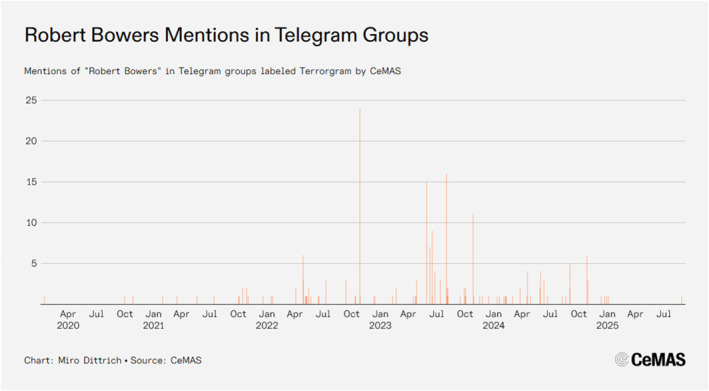
Mentions of Bowers in Telegram groups. (Personal communication with M. Dittrich 2025; graphic used with permission).

At our request, the Center for Monitoring, Analysis and Strategy (CEMAS) conducted a concordance search for “Robert Bowers” in a corpus of over 300 Terrorgram channels and nearly 2 million messages in September 2025 (personal communications, Miro Dittrich, October 2025). Bowers' name appeared in 39 original messages and 208 forwards in CEMAS’ dataset of Terrorgram channels, as well as in 77 original messages and 212 forwards in their dataset of Terrorgram groups (as shown in Figures 7 and 8). The number of mentions spiked during each October (the month of the Pittsburgh synagogue attack) from 2019 through 2024, likely a form of remembrance that is often celebrated on Terrorgram. This analysis highlights that Bowers' attack and influence continue to circulate in these online spaces.

## 6 Discussion

### 6.1 Antisemitic Attacks in the U.S. and Worldwide

Since the October 7, 2023, terror attacks by Hamas, the deadliest day for the Jewish community since the Holocaust, online and offline threats against the Jews have intensified significantly. According to the Global Terrorism Index 2025 (Global Terrorism Index (2025), 36), antisemitic violence and hate crimes have surged across the West, “with attacks on synagogues recorded in Europe, Australia and the US.” The Federal Bureau of Investigation's Hate Crime Statistics Annual Report (2024) noted record high hate crime incidents against the Jewish community in 2024—which accounted for nearly 70% of all religiously motivated hate crimes in the United States—since data collection and reporting began in 1991. Furthermore, the Anti‐Defamation League (Anti‐Defamation League 2025) documented 9354 antisemitic incidents across the United States in 2024, which include assault, harassment, and vandalism, a 344% increase in comparison to the last 5 years and the highest number on record since tracking commenced in 1979.

In 2025 alone, there were several targeted attacks against Jewish places of worship, institutions or celebrations. On May 21, 2025, two staff members from the Israeli Embassy were shot and killed outside the Capital Jewish Museum in Washington, D.C., after attending an annual Young Diplomats reception (United States Attorney's Office, 2025). On June 1, 2025, a group of mostly Jewish individuals who gathered in support of the release of hostages held by Hamas were attacked with gasoline and several Molotov cocktails in Boulder, Colorado (Department of Justice 2025). On October 2, 2025, a terrorist attack occurred on Yom Kippur at a synagogue in Manchester, United Kingdom, when a lone offender conducted a vehicle ramming into pedestrians and stabbed worshippers at the Heaton Park Hebrew Congregation, which killed three—including the perpetrator—and injured three (Counter Terrorism Policing 2025). And on December 14, 2025, an antisemitic terrorist attack inspired by the Islamic State (see Stern and Berger 2015) occurred during a Hanukkah lighting at Bondi Beach in Sydney. The act of violence was carried out in the form of a mass shooting by two perpetrators—a father and son—killing 15 and injuring dozens more. Several improvised explosive devices (IEDs) were located and removed from a vehicle close to the targeted scene.

Conspiracy theory, the most elemental of paranoid fixations, finds common ground historically and contemporaneously on both the extreme left and right: Jihadists share antisemitic beliefs with militant right white supremacists, including Christian nationalists. For example, here is an excerpt from the gab.com website, written by its founder Andrew Torba, 7 years after Bowers' post just prior to the TOL massacre:

But there is something darker still. The Gospel of John records that Jesus was in Jerusalem during the Feast of Dedication—Hanukkah—when the Jews surrounded Him in the temple courts and demanded He tell them plainly if He was the Christ. When He answered, declaring “I and the Father are one,” they picked up stones to kill Him (John 10: 22–31). Hanukkah, in the scriptural record, is the feast during which the Jews attempted to murder the Son of God for declaring His divinity. This is what Christians celebrate when they light the menorah and wish their Jewish neighbors a happy Hanukkah. They celebrate the feast of Christ’s rejection. They celebrate the day stones were gathered to kill Him. They celebrate a temple that God Himself destroyed as judgment for that rejection.

(Torba 2025)

### 6.2 Threat Assessment of the Individual Targeted Attacker

The secrecy with which Bowers planned and prepared for his attack highlights the importance of the complementarity of both strategic prevention and tactical interruption. Strategic prevention is the domain of behavioral threat assessment and management teams (BTAM), optimally composed of professionals from different disciplines, such as law enforcement and mental health; they regularly meet to assess credible threats on the horizon, and take steps to mitigate those threats (Amman et al. 2017; Meloy and Hoffmann 2021). Tactical interruption includes but is not limited to perimeter security and can range from body protection of a public figure to physical security of a potential target, such as a synagogue or a school. The complementarity is found in their dynamic relationship: the effectiveness of BTAM will reduce the frequency of a completely unforeseen attack, while tactical interruption will defend against the lone actor who remains undetected as a credible threat until the moment of his assault.

Along with the utility of BTAM teams is the necessity of using structured professional judgment instruments to assess risk. The use of both *teams and tools* eliminates a twofold problem: the traditional use of only one person to carry the burden of threat assessment and management in an organization, which furthermore heightens the probability of *ipse dixit,* the subjective opinion of one person determining the credibility of a threat based only upon their personal experiences, for example, “I am a police officer, I know.” As mentioned earlier, Viljoen et al. (2024) have recently demonstrated in a large meta‐analysis of studies that instruments are superior to subjective professional judgment in accuracy of risk assessment. This is the largest study to date of a replicated finding over the past several decades.

In the Bowers case, the retrospective analysis utilizing such an instrument, the TRAP‐18, indicates that he was almost identical to other lone‐actor terrorists who have been motivated by different extremist beliefs (Meloy and Gill 2016; Böckler et al. 2021; Vargen and Challacombe 2022) in both his distal characteristics as well as his proximal warning behaviors. Although such a retrospective case analysis does not predict outcome, it contributes to the substantial literature on the TRAP‐18 as a reliable and valid instrument for the assessment of targeted risk (C. S. Allely and Wicks 2022). Bastiaens et al. (2025) have recently proposed to the counterterrorism community a narrow focus on behavioral warning signals for the prevention of targeted violence, an approach advanced by the TRAP‐18 over the past decade.

The Bowers case likewise illustrates the importance of simultaneous investigation of a person of concern both *online and on‐the‐ground*. Given the global ubiquity of the internet and social media, the default assumption among all BTAM efforts should be that the person of concern is leaving behavioral crumbs in both cyberspace and terrestrial terrains. The absence of personnel to investigate his online and offline activity will unwittingly generate *lacunae* of understanding that may be critical for assessment and prevention.

The “pathway to violence” is an original construct in threat assessment and management that has withstood the rigors of both operational use and research challenges (Fein et al. 1995; Calhoun and Weston 2003). Less well understood is the tempo or speed of movement on the pathway of a person of concern. In the case of Bowers, fixation appeared early in his radicalization and energy burst came just prior to his attack—in accordance with research findings (Meloy and Rahman 2020; Kupper and Meloy 2023; Meloy and Kupper 2025). BTAM investigations are wise to answer three questions during the initial stages of a new case: Is the subject on a pathway to violence? If so, where are they on the pathway? And finally, how fast are they moving on the pathway? Research studies related to the latter question are rare (Silver et al. 2018; Brown et al. 2026; Meloy et al. 2021) and deserve further attention. If there is no discernible pathway behavior following a thorough investigation, the case is unlikely to be a high or imminent risk *at the present time*.

A final note in the threat assessment and management of subjects of concern for lone‐actor terrorism is the importance of disaggregation. What is it that we are exactly trying to prevent? Is this person, if they continue on their current pathway, likely to be a terrorist actor, a facilitator of someone who will actually carry out the violence, or merely a supporter of those who do engage in targeted violence in the service of a particular cause or ideology? The distal characteristics within the TRAP‐18 offer little in the way of disaggregation, and even the first proximal warning behaviors, such as fixation, cannot foresee the eventual outcome of the case. Later proximal warning behaviors, in particular pathway, last resort, energy burst and identification, can detect the violent signal, discern motivation and may often predict the outcome. Disaggregation in terrorism research has been studied from a variety of perspectives (Clemmow et al. 2022; Gill and Corner 2013; Perliger et al. 2016).

### 6.3 The Macro Environment: Detecting and Managing Contagion and Copycat Effects Within the Current Political and Social Noise

Meloy (Kupper et al. 2022) proposed a distinction between the terms *contagion* and *copycat* in the targeted violence and prevention community. *Contagion* is an acute period, usually 2 weeks, in which a highly publicized mass attack leads to an increase above baseline in subsequent attacks through imitation of the act. This idea is based upon the work of Towers et al. (2015). *Copycat*, on the other hand, is a chronic phenomenon, usually spanning months if not years, in which both the act and the actor are imitated; when aggregated, such copycat events become a subcultural script, most often accelerated through the rhetoric of an online community. The subsequent online dispersion of information and adulation afforded Bowers is a clear example of the copycat phenomena: online identification with a violent and grandiose murderer becomes future on‐the‐ground terror directed toward other civilians.

How do we mitigate the macro risks of the lone actor? First is the acceptance that government has a role in the regulation of free enterprise. As President John F. Kennedy stated many years ago, “we should not depend solely on a free competitive economy to curb its own abuses” (Kennedy 1952). This may be viewed as a platitude by some, but digging into the weeds is necessary: How does the government balance the public welfare with first amendment protections of free speech? Should Section 230 of the Communications Act be changed to hold companies such as Meta and Google responsible for the content which they disperse? How can a moral core be sustained by the so‐called Magnificent Seven in their pursuit of Artificial General Intelligence (Hao 2025)? How can evolving community standards keep pace with the LLMs (large language models) of AI? When will the titans of the tech industries recognize that with every technological innovation comes new methods of criminality, and that it is grossly naïve to assume that human nature is only limited to benign and prosocial behavior?

Returning to the task at hand—the prevention of extremist attacks—we close with an important statement from The Soufan Group:

The United States government needs to be concerned about all types of ideologically and politically motivated violence, no matter which side of the spectrum it emanates from. The [Turtle Island Liberation Front] plot could be the sign of more terrorism from the far‐left in the coming year, but other threats remain on the horizon, including that posed by the persistence of jihadist groups, including the Islamic State. Picking and choosing which types of terrorism to combat will make all Americans less safe, and law enforcement and the intelligence community need to remain focused on the tactics, techniques, and procedures of these groups, as well as what grievances are motivating their turn toward violence and extremism

(The Soufan Center 2025).

## 7 Conclusion

Robert Bowers' mass murder was not prevented because nobody was looking at him. Carefully following the “gray man” script, he avoided detection of his warning behaviors prior to attack. However, he was demonstrating seven of 10 distal characteristics on the TRAP‐18. This would have warranted deeper scrutiny had they been detected by a threat assessment professional trained in the instrument. Though Bowers' PWBs were hidden from view in the pre‐attack period, he demonstrated six of eight, including all four of those PWBs most closely associated with high or imminent risk: pathway, identification, energy burst, and last resort.

Having carried out the most lethal antisemitic attack in the U.S. to date, he has become an inspiration to other successful and would‐be lone‐actor terrorists and mass murderers. Conducting a linguistic assessment of social media and other expressive activity of persons of concern should be considered an integral part of a TRAP‐18 analysis by threat assessment professionals. Much can be discerned or at least hypothesized by understanding from whom persons of concern draw their inspiration and education. With antisemitic attacks continuing to rise, retrospective case analyses such as this one will continue to inform and drive forward the body of knowledge dedicated to preventing targeted attacks.

## Author Contributions

M.A., J.K., and J.R.M. jointly conceptualized the article, contributed analysis, and wrote and edited drafts. Author M.A. led writing and organization.

## Funding

The authors have nothing to report.

## Ethics Statement

The authors confirm that the data utilized in this study are open‐source and publicly available, therefore, formal institutional review board (IRB) approval was not required. Dr. Meloy derives income from the marketing of both the TRAP‐18 manual and code sheets, and training on the instrument.

## Data Availability

The data that support the findings of this study are available on request from the corresponding author, M.A.

## Endnotes

^1^
As it turned out, this was a miscalculation. While patrol officers did respond initially, Bowers was subsequently confronted by SWAT officers, who wore body armor capable of withstanding strikes from 0.357 rounds.

^2^
Dr. Meloy derives income from the marketing of both the TRAP‐18 manual and code sheets, and training on the instrument.

^3^
TRAP‐18 Manual version 2.0., Multi‐Health Systems (mhs.com).

^4^
Bowers told Dr. Park Dietz that the secondary target was a JFC—Jewish Federation Center, while he told Dr. Richard Rogers the secondary target was a JCC—Jewish Community Center. There was one of each in proximity to TOL, so it remains unclear which was the true secondary target, or if Bowers was certain himself.
